# Closing the Gap: a Site-Developed Quality Improvement Project to Optimize the Early Care of Pregnant Veterans

**DOI:** 10.1007/s11606-022-07593-3

**Published:** 2022-08-30

**Authors:** Zena Tamar White, Simone Kanter, Karuna Ahuja

**Affiliations:** VA San Diego Healthcare System, San Diego, CA USA

## **INTRODUCTION**

From FY00 to FY15, the Veterans Health Administration (VHA) saw a 14-fold increase (FY00: 260; FY15: 3756) in deliveries among pregnant veterans.^1^ The number of pregnant veterans is expected to rise as more young women enter the military. In the past five years, VA San Diego Healthcare System (VASDHS) provided and coordinated care for about 1500 pregnant veterans.

Although they receive their maternity care in the community, pregnant veterans continue to access the VA for their primary and specialty care needs.^2^ VA primary care providers (PCPs) are the first point of contact for pregnant veterans and serve a pivotal role in care coordination including referrals within VA and the community.^3,4^ Mental health conditions (depression, anxiety, PTSD) among women veterans often worsen during pregnancy and may require collaboration with mental health specialists for closer monitoring.^5^

A detailed maternity care consult placed by a PCP is required for veterans in VASDHS to receive prenatal care in the community. This consult specifically asks about the patient’s medical, obstetric, mental health, social history, and current medications. During the interval between a maternity care consult and the veteran’s first prenatal appointment which is often 4–6 weeks, pregnant veterans may seek advice and medical care from their PCPs. Addressing essential issues early on that impact the health of a pregnancy including medication reconciliation, mental health concerns, substance use, and personal safety is critical to provide optimal care to pregnant veterans.^6,7^

At VASDHS, the authors designed a survey as the first part of a quality improvement project to (1) assess PCPs’ screening and care practices towards pregnant veterans and (2) utilize results to further educate PCPs on their roles in the maternity care referral process and caring for pregnant veterans.

## METHODS

The authors designed a 15-item paper survey of closed and open-ended questions.

The survey combined questions taken directly from the VA National Maternity Care consult with additional questions related to the mental health and safety of pregnant veterans. To achieve a robust response rate, the authors distributed this survey during VASDHS Primary Care Grand Rounds in October 2019. Surveys were completed voluntarily and anonymously. Soon thereafter, University of California, San Diego, Internal Medicine Residents who are considered PCPs for their patients also completed the surveys during their assigned VA continuity clinics.

## RESULTS

Sixty-one out of seventy surveys (87%) were completed, forty-six from PCPs and twenty-five from Internal Medicine Residents. Regarding the maternity care referral process and primary care practices, 31% of PCPs never and 10% rarely entered a progress note in the medical record documenting their discussion regarding new pregnancy diagnosis, review of screening questions, or care plan with their patient. Table 1 depicts the percentage of PCPs reviewing early health-related pregnancy topics during the maternity care referral process. Over half of PCPs sometimes never inquire about mental health problems in early pregnancy. Eighty percent of PCPs expressed interest in receiving further education about the maternity care referral process and provision of care during early pregnancy.

**Table 1:** VASDHS Primary Care Provider Screening Practices During Maternity Care Referral. *N* = 61 PCPs Who Completed Survey

**Pregnancy care topics**	**Frequency at which PCPs review pregnancy care topics**					
	Always (%)	Often (%)	Sometimes (%)	Rarely (%)	Never (%)	Nonresponse (%)
Pregnancy desired	41	16	20	5	16	2
Medication reconciliation	62	7	10	3	13	5
Prenatal vitamins	52	20	3	3	20	2
Intimate partner violence	20	20	27	15	15	3
Substance use	67	12	2	3	13	3
Military sexual trauma	20	13	29	11	25	2
Mental health problems	24	25	20	11	20	0
Referral to mental health (if indicated)	25	41	11	2	16	5

## DISCUSSION

**The survey identified a significant number of PCPs who never review critical health screening topics in early pregnancy**. This represents a missed opportunity to identify health concerns and risks among pregnant veterans which may require early intervention and coordination with specialists. Given the high prevalence of depression, anxiety, and PTSD which often worsen during pregnancy and post-partum period, identification of mental health concerns and collaboration with mental health providers is critical to optimize outcomes for the veteran and infant.

*In response to the survey results, the authors delivered a formal presentation in June 2021 during Primary Care Grand Rounds highlighting the role of the PCP in early stages of pregnancy including screening and co-managing conditions that may impact the health of pregnancy and post-partum period.* Ongoing educational activities in this area are in development. The authors plan to resurvey VA Primary Care Providers in the next year to determine if these educational initiatives improve screening, knowledge, and care practices for pregnant veterans.
